# Synthesis of cyanopyridine based conjugated polymer

**DOI:** 10.1016/j.dib.2016.04.007

**Published:** 2016-04-11

**Authors:** B. Hemavathi, T.N. Ahipa, Saju Pillai, Ranjith Krishna Pai

**Affiliations:** a Technology Mission Division, Department of Science and Technology (DST), Ministry of Science and Technology, Government of India, Technology Bhavan, New Mehrauli Road, New Delhi −110016110016, India; b CNMS, Jain University, Jain Global Campus, Bangalore – 562112, India; c Materials Science and Technology Division & Academy of Scientific and Innovative Research (AcSIR), CSIR-National Institute for Interdisciplinary Science and Technology (CSIR-NIIST), Thiruvananthapuram 695019, Kerala, India

## Abstract

This data file contains the detailed synthetic procedure for the synthesis of two new cyanopyridine based conjugated polymer **P1** and **P2** along with the synthesis of its monomers. The synthesised polymers can be used for electroluminescence and photovoltaic (PV) application. The physical data of the polymers are provided in this data file along with the morphological data of the polymer thin films. The data provided here are in association with the research article entitled ‘Cyanopyridine based conjugated polymer-synthesis and characterisation’ (Hemavathi et al., 2015) [3].

**Specifications Table**

**Table:** 

Subject area	*Chemistry*
More specific subject area	*Synthesis of conjugated polymers*
Type of data	*Graph, image*
How data was acquired	*NMR, UV–vis, CV, AFM*
Data format	*Analyzed*
Experimental factors	*The materials required for polymer synthesis are either synthesized according to available standard procedure or directly procured*
Experimental features	*The synthesized compounds are analyzed for their chemical, photophysical, thermal, and electrochemical properties.*
Data source location	*Bangalore, India*
Data accessibility	*Data are provided with this article*

**Value of the data**

• The data presented here is essentially aimed at reproducing the synthetic route of the new conjugated cyanopyridine based polymer.
• The data provides an opportunity to synthesis derivatives of the cyanopyridine based polymers.
• The data provided here will be a reference value for the comparison new experimental data for the synthesis of conjugated polymers.

## 1 Data

The NMR data was obtained at 400 MHz with Jeol AV-III400 (L) spectrometer using deuterated CDCl_3_ or DMSO-*d*_6_ as solvents. The chemical shift (δ) is represented with part per million (ppm). The UV–vis and photoluminescence data were obtained for polymer solution in CHCl_3_. The energy levels were investigated with Electrochemical (CV). The AFM data were obtained with Bruker Instrument for the polymer thin film.

## 2 Experimental design, materials and methods

The polymers were designed to be synthesised with Heck polymerisation technique. The synthesised polymers were after purification were analysed for chemical, photophysical, electrochemical and thermal properties. The reagents and organic solvents were procured from Spectrochem, and used as received without much purification. Compounds **2** and **3** were procured from Spectrochem and compound **1** was synthesised according to the literature [1].

### 2.1 Synthesis of monomers and polymers

#### 2.1.1 Synthesis of 1,3-*Bis*(4-bromophenyl)prop-2-en-1-one (**4**)

**Scheme 1:**
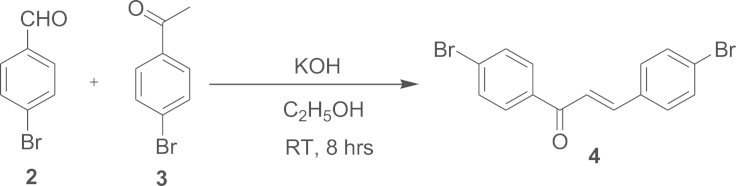
Synthesis of compound **4**.

**Scheme 2:**
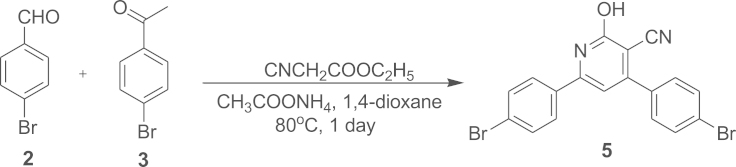
Synthesis of compound **5**.

**Scheme 3:**
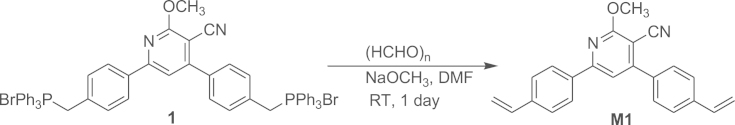
Synthesis of compound **M1**.

**Scheme 4:**
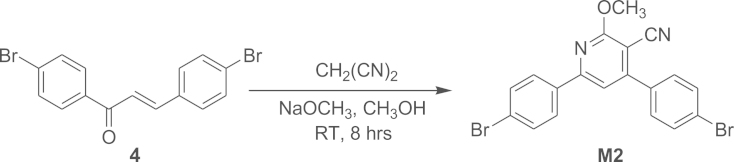
Synthesis of compound **M2.**.

**Scheme 5:**
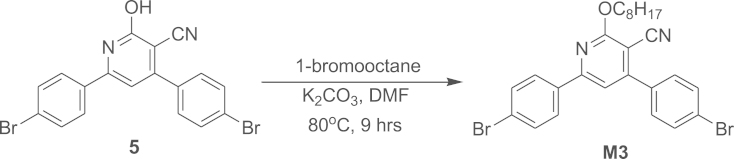
Synthesis of compound **M3**.

The compound **4** was synthesised through Claisen–Schmidth reaction from 4-bromobenzaldehyde (compound **2**) and 4-bromoacetophenone (compound **3**) Scheme 1, Scheme 2, Scheme 3, Scheme 4, Scheme 5

Compound **2**, (2 g, 10.8 mmol) and compound **3**, (2.15 g, 10.8 mmol) were dissolved in 15 mL ethanol. Aqueous solution of potassium hydroxide (3.24 mmol) prepared by dissolving 0.72 g in 2 ml of water was added with constant stirring, and kept the reaction on for 8 h at room temperature. The product obtained as precipitate in the reaction mixture was filtered and washed with 10 mL ethanol to remove the impurities. The compound is then recrystallised from 1:1 ratio of ethanol and chloroform mixture to obtain a pale yellow product **4** (3.722 g, 94%). Melting point, *T*_m_=186.57 °C. ATR-IR (cm^−1^): 810, 998, 1180, 1595, 1654. ^1^H NMR (400 MHz, CDCl_3_, d/ppm): 7.43 (S, 1H), 7.49 (d, *J*=1.6 Hz, 1H), 7.51 (d, *J*=2 Hz, 1H), 7.55 (d, *J*=2 Hz, 1H), 7.57 (d, *J*=2 Hz, 1H), 7.64 (d, *J*=2 Hz, 1H), 7.66 (d, *J*=2 Hz, 1H), 7.72 (s, 1H), 7.88 (d, *J*=2 Hz, 1H), 7.89 (d, *J*=2 Hz, 1H). ESI-MS/MS (C_15_H_10_Br_2_O), [M+Na]^+^: calcd, 389.05; found, 389.2.

#### 2.1.2 Synthesis of 4,6-Bis(4-bromophenyl)-2-hydroxypyridine-3-carbonitrile (**5**)

Compound **5** was synthesised from one pot synthesis of compounds **2** and **3**.

Compound **2**, 4-bromobenzaldehyde (2 g, 10.8 mmol) and compound **3**, 4-bromoacetophenone (2.15 g, 10.8 mmol) were dissolved in 35 mL of 1,4-dioxane. Ammonium acetate (6.6 g, 85.7 mmol) was added followed with 1.5 mL of ethyl cyanoacetate (12.9 mmol) with constant stirring and kept the reaction at 80 °C for 24 h. The reaction mixture was cooled and poured into 100 mL of distilled water and stirred for 10 min. The obtained precipitate was filtered and the filtrate was thoroughly washed with 1,4-dioxane to remove the impurity and dried to afford a white product **5** (1.66 g, 36%). Melting point, *T*_m_=324.89 °C. ATR-IR (cm^−1^): 810, 1004, 1230, 1495, 1635, 2219 (–CN). ^1^H NMR (400 MHz, CDCl_3_, d/ppm): 3.56 (s, 1H), 7.68 (d, *J*=2 Hz, 2H), 7.70 (d, *J*=2 Hz, 2H), 7.73 (d, *J*=2 Hz, 1H), 7.75 (d, *J*=2 Hz, 2H), 7.77 (t, *J*=2 Hz, 2H), 7.79 (d, *J*=2 Hz, 1H). ESI-MS/MS (C_18_H_10_Br_2_N_2_O), [M+H]^+^: calcd, 431.09; found, 431.2.

#### 2.1.3 Synthesis of 2-Methoxy-4,6-bis (4-vinylphenyl) pyridine-3-carbonitrile (**M1**)

Monomer **M1** was synthesised via Wittig reaction from compound **1** in presence of paraformaldehyde.

Compound **1** (1 g, 1.003 mmol) was dissolved in 8 mL *N*,*N*-dimethylfarmamide (DMF) and 67.90 mg of paraformaldehyde (2.204 mmol) was added and stirred the mixture. Further, sodium methoxide solution (prepared by adding 103 mg of sodium (4.904 mmol) in 4 mL of methanol) was added slowly into it with constant stirring and allowed to stand for 2 days at room temperature. The reaction mixture was poured into 30 mL of distilled water and the precipitate was filtered. The product was then sonicated with 15 mL of methanol for 10 min to remove the impurity and filtered and dried to afford bright yellow product **M1** (250 mg, 73%). Melting point, *T*_m_=196.87 °C. ATR-IR (cm^−1^): 1106, 1436, 1571, 1659, 2218 (–CN). ^1^H NMR (400 MHz, CDCl_3_, d/ppm): 4.16 (m, *J*=5.2 Hz, 3H), 7.41 (m, *J*=7.6 Hz, 2H), 7.53 (m, *J*=12 Hz, 2H), 7.65 (m, *J*=8 Hz, 2H), 7.82 (m, *J*=8.4 Hz, 5H), 7.91 (m, *J*=12 Hz, 2H), 8.33 (m, *J*=4.8 Hz, 2H). ESI-MS/MS (C_23_H_18_N_2_O), [M+H]^+^: calcd, 339.40; found, 339.2.

#### 2.1.4 Synthesis of 4,6-Bis(4-bromophenyl)-2-methoxypyridine-3-carbonitrile (M2)

Compound **4** was cyclised with malononitrile to obtain comonomer **M2.**

Compound **4** (3.5 g, 9.560 mmol) was dissolved in 5 mL of methanol and 15 mL of sodiummethoxide (190 mmol) solution (prepared by dissolving 4 g of sodium in 15 mL methanol) was added with constant stirring. 1 mL of malononitrile was added with a micro-syringe to the reaction mixture at room temperature with constant stirring and allowed the reaction for 8 h at room temperature. The product precipitates out from the reaction mixture and the precipitate was filtered and washed with methanol to remove the impurities and then dried to afford creamy yellow coloured product **M2** (2.008 g, 47%). Melting point, *T*_m_=174.13 °C. ATR-IR (cm^−1^): 817, 1005, 1366, 1539, 2217 (–CN). ^1^H NMR (400 MHz, CDCl_3_, d/ppm): 3.30 (s, 3H), 7.52 (m, *J*=1.6 Hz, 1H), 7.53 (m, *J*=2.8 Hz, 2H), 7.55 (m, *J*=2.8 Hz, 2H), 7.60 (m, *J*=1.6 Hz, 2H), 7.63 (m, *J*=1.2 Hz, 2H). ESI-MS/MS (C_19_H_12_ Br_2_N_2_O), [M+CH3OH]^+^: calcd, 473.9; found, 473.0.

#### 2.1.5 Synthesis of 4, 6-Bis (4-bromophenyl)-2-(octyloxy) pyridine-3-carbonitrile (**M3**)

Alkylation of Compound **4** with 1-bromooctane affords monomer **M3**.

Compound **5** (0.33 g, 0.767 mmol) was dissolved in 8 mL of DMF. 1-bromooctane (0.15 mL, 0.777 mmol) was then added, followed by 148 mg of potassium carbonate (1.072 mmol) and the reaction mixture was stirred at 90 °C for overnight. The reaction mixture was cooled and poured into ice cold water with constant stirring. The obtained precipitate was filtered, washed with water and dried to obtain creamy yellow product **M3** (280 mg, 75%). Melting point, *T*_m_=79.16 °C. ATR-IR (cm^−1^): 820, 998, 1348, 1540, 2219 (–CN), 2918. ^1^H NMR (400 MHz, CDCl_3_, d/ppm): 0.88 (t, *J*=4.8 Hz, 3 H), 1.29 (m, *J*=6.4 Hz, 4H), 1.39 (m, *J*=4 Hz, 4H), 1.519 (m, *J*=7.2 Hz, 4H), 1.89 (m, *J*=6.8 Hz, 2H), 4.576 (t, *J*=6.4 Hz, 2H), 7.26 (s, 1H) 7.52 (d, *J*=6.8 Hz, 2H), 7.61 (d, *J*=2 Hz, 1H), 7.63 (d, *J*=2 Hz, 1H), 7.66 (d, *J*=2 Hz, 1H), 7.68 (d, *J*=2 Hz, 1H), 7.92 (d, *J*=2 Hz, 1H), 7.94 (d, *J*=2 Hz, 1H). ESI-MS/MS (C26H26Br2N2O), [M+H]^+^: calcd, 543.31; found, 543.1.

### 2.2 Synthesis of polymers **P1** and **P2**

**Scheme 6:**
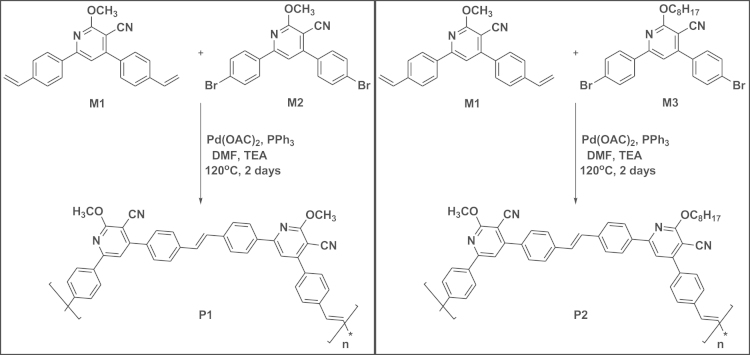
Synthesis of polymers **P1** and **P2**.

Synthetic route for the preparation of copolymer **P1** and **P2** is shown in Scheme 6.

Polymer **P1** was synthesised from the reaction of the monomer **M2** with monomer **M1** under nitrogen atmosphere via Heck coupling reaction. The monomers, **M1** (0.1 g, 0.295 mmol) and **M2** (0.1312 g, 0.297 mmol) were dissolved in 8 mL DMF and 7 mL triethylamine (TEA) solvents and stirred purged with nitrogen gas for 15 min. To this, triphenylphosphine (250 mg, 0.953 mmol) was added and again purged with nitrogen gas for 10 min. Further, catalytic amount (0.1 eq.) of palladium (II) acetate (Pd(OAc)_2_) was added to the reaction mixture under inert atmosphere and stirred at 120 °C for 2 days. The reaction mixture was cooled and poured into a beaker containing 40 mL methanol and the obtained precipitate was filtered. The crude polymer was then dissolved in chloroform and undissolved impurities are removed by filtration. The polymer was then precipitated in methanol and washed with acetone (to remove oligomers) to afford dark yellow product **P1** (0.099 g, 53%). ATR-IR (cm^−1^): 696, 826, 963 (*trans*-vinylene), 1008, 1360, 1589, 2222 (–CN). ^1^H NMR (400 MHz, CDCl_3_, d/ppm): 4.24 (b, 3H), 7.26 (b, 2H), 7.54 (b, 4H), 7.69 (b, 5H), 8.13 (b, 2H).

Copolymer **P2** was synthesised from the reaction of the monomer M3 with monomer M1 under nitrogen atmosphere via Heck coupling reaction. The monomers, M1 (0.1 g, 0.295 mmol) and M3 (0.16 g, 0.295 mmol) were dissolved in 8 mL DMF and 7 mL triethylamine (TEA) solvents and stirred under nitrogen atmosphere for 15 min. To this, triphenylphosphine (250 mg, 0.953 mmol) was added and again purged with nitrogen gas for 10 min. Further, catalytic amount (0.1 eq.) of palladium (II) acetate (Pd(OAc)_2_) was added to the reaction mixture under inert atmosphere and stirred at 120 °C for 2 days. The reaction mixture was cooled and poured into a beaker containing 40 mL methanol and the obtained precipitate was filtered. The crude polymer was then dissolved in chloroform and undissolved impurities are removed by filtration. The polymer was then precipitated in methanol and washed with acetone (to remove oligomers) to afford dark yellow product **P2** (0.125 g, 57%). ATR-IR (cm^−1^): 832, 966 (*trans*-vinylene), 1004, 1147, 1358, 1576, 2222 (–CN), 2946. ^1^H NMR (400 MHz, CDCl3, d/ppm): 0.88 (b, 3H), 1.31 (b, 10H), 1.91 (b, 2H), 4.24 (b, 3H), 4.61 (b, 2H), 7.27 (b, 6H), 7.54 (b, 4H), 7.71 (b, 8H), 8.15 (b, 4H). *M*_n_=841,401 g/mol, *M*_w_=941,465 g/mol, PDI= 1.12.

## 3 UV–visible spectroscopy

**Fig. 1:**
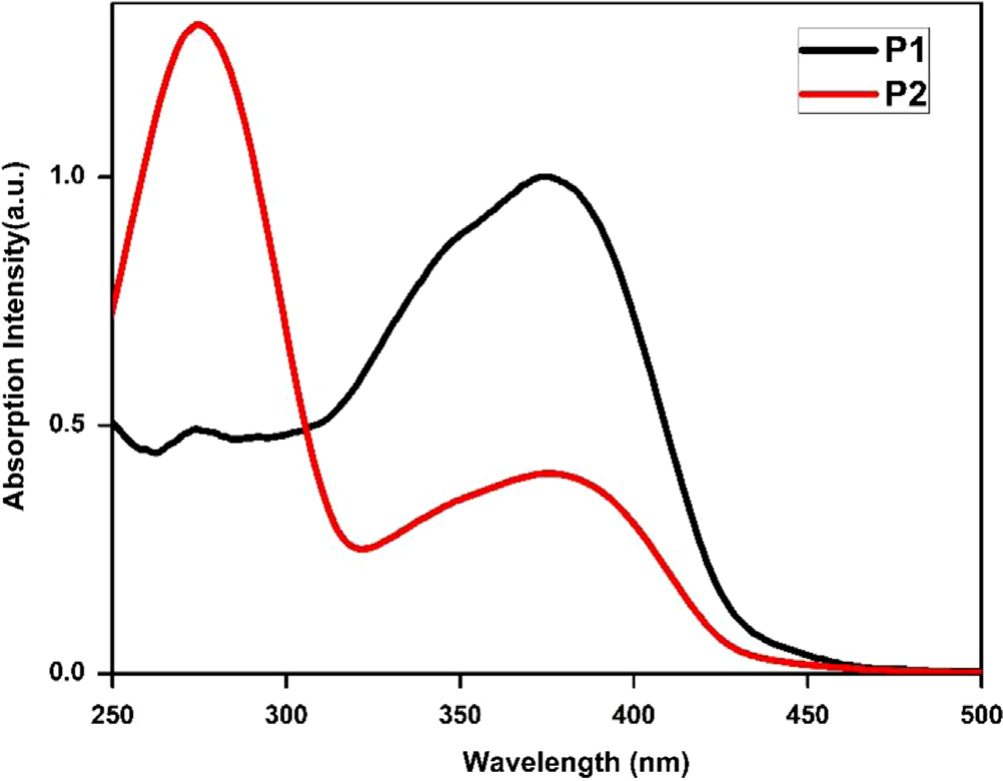
Absorption spectra of **P1** and **P2** in solution.

The UV–vis spectra were obtained using a Shimadzu UV-1800 spectrophotometer. A stock solution of 2 mg/mL of the polymer in CHCl_3_ was prepared. 15 µL of this stock solution was diluted with 3 mL of CHCl_3_ for the analysis. The UV–vis spectra of polymers **P1** and **P2** in solution and film are depicted in Fig. 1.

## 4 Cyclic voltammetry

**Fig. 2:**
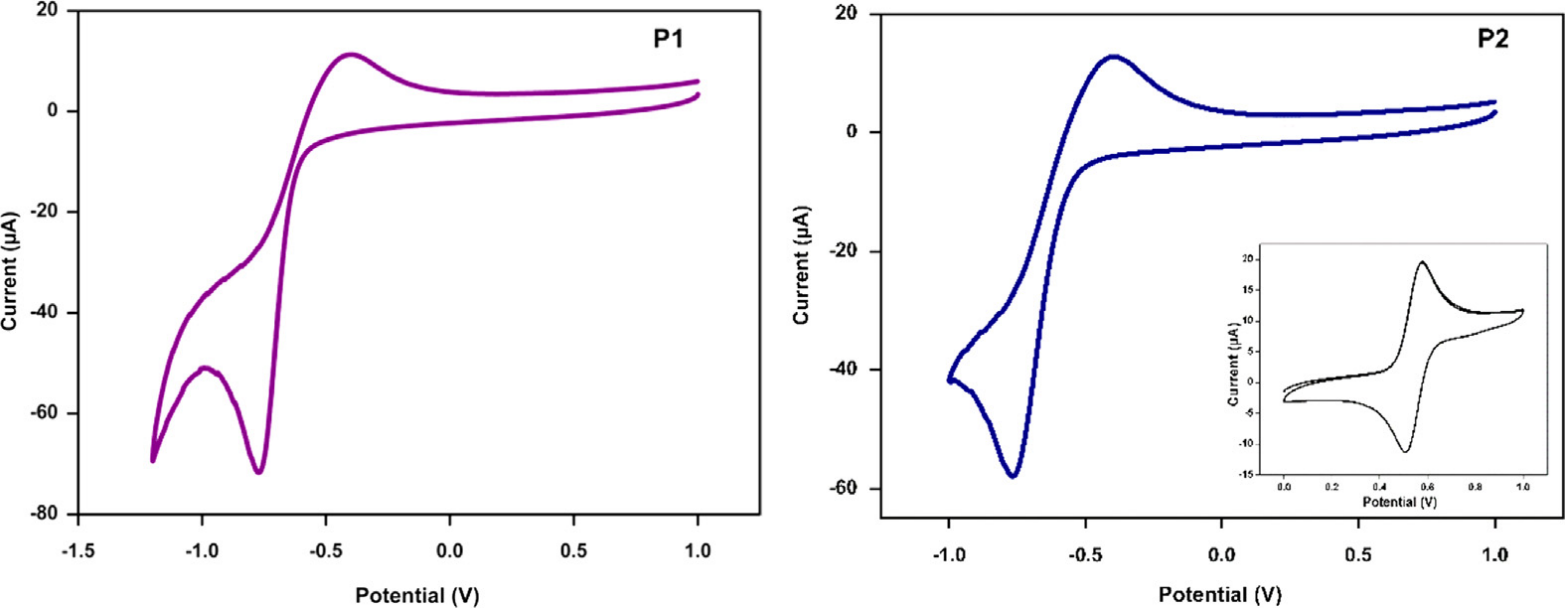
Voltammograms of **P1** and **P2**.

The CV was recorded for polymer coated on glassy carbon from chloroform solution (2 mg/ml) with Ag/AgCl as reference electrode and calibrated against Fc/Fc^+^ redox couple for which ionisation energy is taken to be 4.80 eV [2]. The LUMO of the polymers were calculated with *E*_LUMO_=−*e* (*E*_red_+4.33) (ionisation potential for Fc/Fc^+^ measured was 0.47 V vs Ag/AgCl). The voltammograms for the polymers are as shown in Fig. 2.

## 5 Atomic force microscopy

**Fig. 3:**
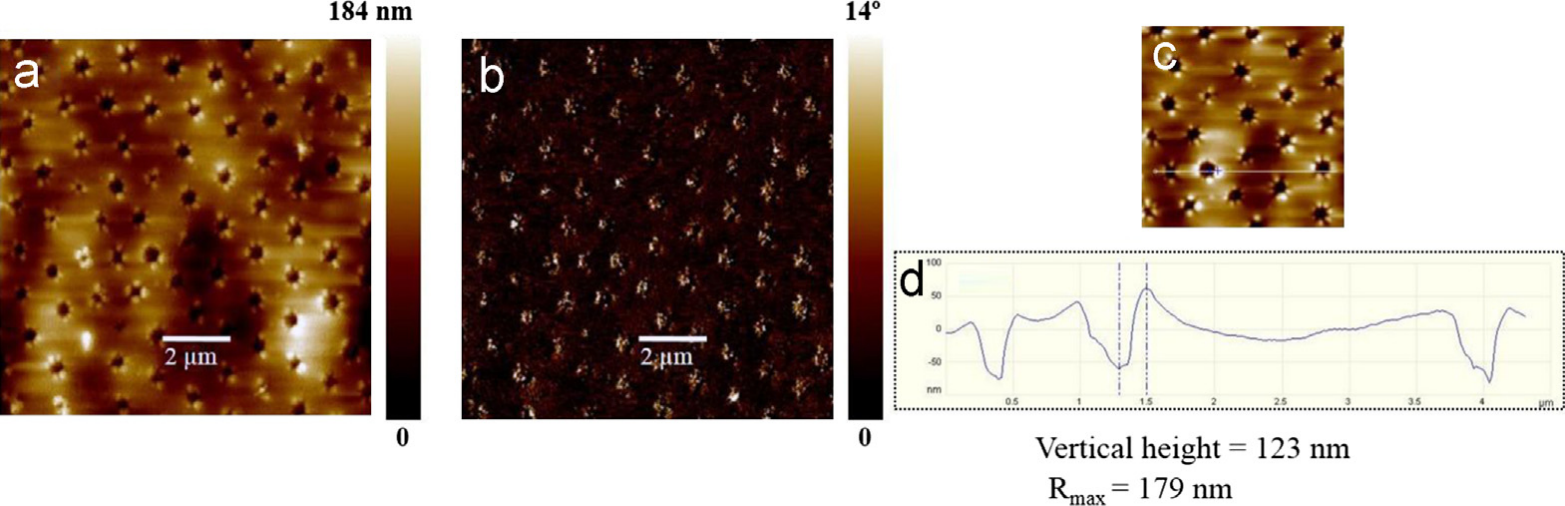
AFM images of **P1** (a) height image, (b) phase image (c) corresponds to higher-magnification image and figure (d) is corresponding vertical height images with position of vertical height is marked in (c).

**Fig. 4:**
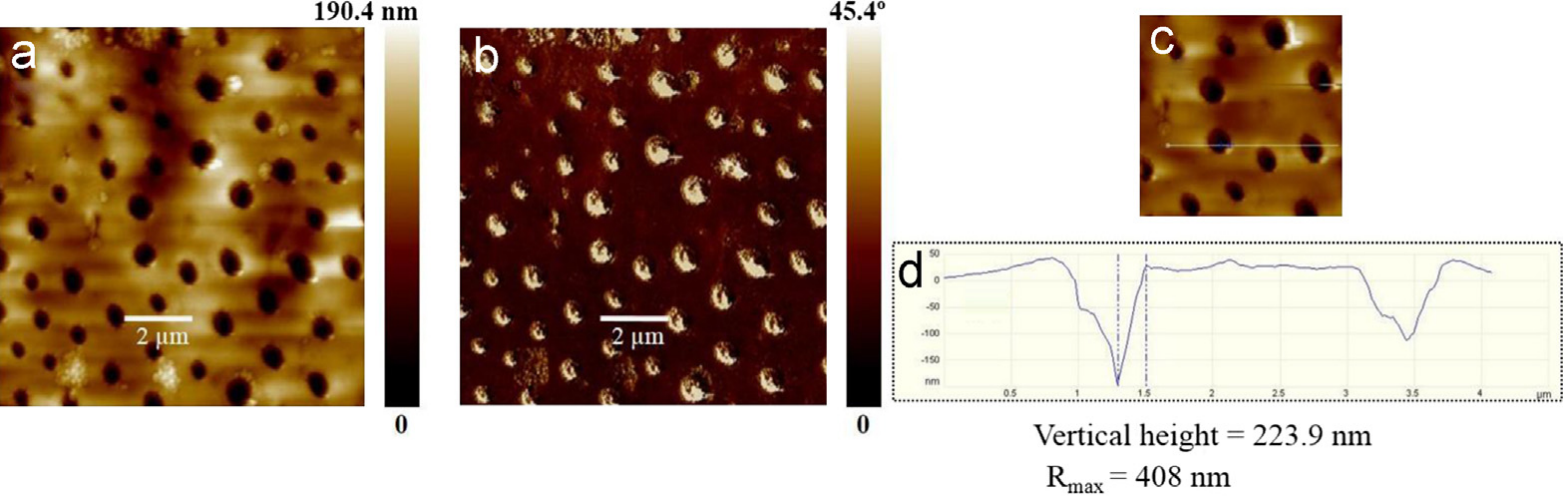
AFM images of **P2** (a) height image, (b) phase image (c) corresponds to higher-magnification image and figure (d) is corresponding vertical height images with position of vertical height is marked in (c).

Atomic force microscopy (AFM) imaging was performed under dry conditions at room temperature (22±2 °C) using MultiMode 8 AFM equipped with NanoScope V controller (Bruker, Santa Barbara, CA, USA). Si cantilevers (NSG 01, NT-MDT) with a typical radius of curvature of approximately 10 nm were used. The force constants of AFM probe in the range of 2.5–10 N/m and with resonance frequency in the range of 120–180 kHz. The scan rate used was 1 Hz. The samples for AFM measurements were prepared by drop-casting 30 µL of MST samples onto a fleshly cleaved mica sheet and dried overnight under vacuum. Raw data were processed offline using Bruker׳s NanoScope Analysis software. Surface roughness was reported both in average roughness values. The AFM images of the polymers **P1** and **P2** are depicted in Fig. 3, Fig. 4 respectively.
