# Supplementary material for: Synthesis of cyanopyridine based conjugated polymer
Source: Data Brief. 2016 Apr 11;7:1314–20. doi: 10.1016/j.dib.2016.04.007 (PMC4842847; doi:10.1016/j.dib.2016.04.007)
Supplement: Supplementary file 1 — Supplementary material [file mmc1.docx]

To:

Alessandro Mottura, PhD
Section Editor
Data in Brief

Dear Sir,

***Sub****: Declaration form – conflict of interest*

Manuscript title: **“Synthesis of Cyanopyridine Based Conjugated Polymer”**for publication in your esteemed journal.

Reference No:  ***DIB-D-15-00402****.*

**I declare no conflict of interest for the above mentioned research work**

Thanking you,

Sincerely,
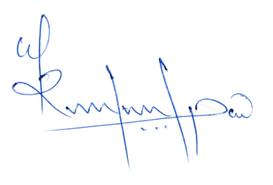


Dr. Ranjith Krishna Pai

Associate Professor

Center for Nano and Material Sciences

Jain University Date: 20/10/2015

Place: Bangalore
